# Iodide of Starch

**Published:** 1880-02

**Authors:** 


### IODIDE OF STARCH.

As a general antidote in poisoning, Dr. Bellini, in a paper read before the Medical Society of Florence, recommends the iodide of starch as an antidote to poison generally. It is free from any disagreeable taste, and has not the irritating properties of iodine, so that it can be administered in large doses.

He has made numerous experiments, and states as a result of these, that at the temperature of the stomach and in the presence of the gastric juice the iodide combines with many of the poisons, forming in some cases insoluble compounds, in others soluble compounds, which are harmless, so long as they are not in too large quantities. He recommends it as safe in all cases where the nature of the poison is unknown, and as especially efficient in cases of poisoning by the alkaloids and alkaline sulphides, by caustic alkalies, by ammonia and especially by those alkaloids with which iodide forms insoluble compounds. In cases of poisoning by salts of lead and mercury, it aids the elimination of these compounds. In cases of acute poisoning, an emetic should be employed soon after the administration.

